# Persistent SARS-CoV-2 PCR Positivity Despite Anti-viral Treatment in Immunodeficient Patients

**DOI:** 10.1007/s10875-023-01504-9

**Published:** 2023-05-06

**Authors:** Michele Chan, Me Me Nay Linn, Thomas O’Hagan, José Afonso Guerra-Assunção, Angie Lackenby, Sarita Workman, Anna Dacre, Siobhan O. Burns, Judith Breuer, Jennifer Hart, Susan Tadros, David M. Lowe

**Affiliations:** 1 Department of Clinical Immunology, Royal Free London NHS Foundation Trust, London, UK; 2 University College London Medical School, London, UK; 3 Institute of Child Health, University College London, London, UK; 4 UK Health Security Agency, London, UK; 5 Institute of Immunity and Transplantation, University College London, Pears Building, Rowland Hill Street, London, NW3 2PP UK; 6 Department of Virology, Royal Free London NHS Foundation Trust, London, UK

**Keywords:** SARS-CoV-2, persistence, COVID-19, antivirals, immune deficiency

## Abstract

**Purpose:**

COVID-19 infection in immunodeficient individuals can result in chronically poor health, persistent or relapsing SARS-CoV-2 PCR positivity, and long-term infectious potential. While clinical trials have demonstrated promising outcomes using anti-SARS-CoV-2 medicines in immunocompetent hosts, their ability to achieve sustained viral clearance in immunodeficient patients remains unknown. We therefore aimed to study long-term virological outcomes in patients treated at our centre.

**Methods:**

We followed up immunocompromised inpatients treated with casirivimab-imdevimab (Ronapreve) between September and December 2021, and immunocompromised patients who received sotrovimab, molnupiravir, nirmatrelvir/ritonavir (Paxlovid), or no treatment from December 2021 to March 2022. Nasopharyngeal swab and sputum samples were obtained either in hospital or in the community until sustained viral clearance, defined as 3 consecutive negative PCR samples, was achieved. Positive samples were sequenced and analysed for mutations of interest.

**Results:**

We observed sustained viral clearance in 71 of 103 patients, none of whom died. Of the 32/103 patients where sustained clearance was not confirmed, 6 died (between 2 and 34 days from treatment). Notably, we observed 25 cases of sputum positivity despite negative nasopharyngeal swab samples, as well as recurrence of SARS-CoV-2 positivity following a negative sample in 12 cases. Patients were then divided into those who cleared within 28 days and those with PCR positivity beyond 28 days. We noted lower B cell counts in the group with persistent PCR positivity (mean (SD) 0.06 (0.10) ×10^9^/L vs 0.22 (0.28) ×10^9^/L, *p* = 0.015) as well as lower IgA (median (IQR) 0.00 (0.00–0.15) g/L vs 0.40 (0.00–0.95) g/L, *p* = 0.001) and IgM (median (IQR) 0.05 (0.00–0.28) g/L vs 0.35 (0.10–1.10) g/L, *p* = 0.005). No differences were seen in CD4+ or CD8+ T cell counts. Antiviral treatment did not impact risk of persistent PCR positivity.

**Conclusion:**

Persistent SARS-CoV-2 PCR positivity is common among immunodeficient individuals, especially those with antibody deficiencies, regardless of anti-viral treatment. Peripheral B cell count and serum IgA and IgM levels are predictors of viral persistence.

**Supplementary Information:**

The online version contains supplementary material available at 10.1007/s10875-023-01504-9.

## Introduction

Coronavirus disease 2019 (COVID-19) can manifest as a persistent or relapsing infection in immunodeficient hosts. This has shown to result in long-term infectious potential [1], chronically poorer health [2], and even fatal outcomes [3]. Patients with primary and secondary immunodeficiencies experience greater morbidity and mortality [4, 5]. Persistent SARS-CoV-2 infection in such individuals poses the additional concern of viral mutation towards immunological escape and drug resistance [6, 7]. Extensive efforts have therefore been made on finding the optimal treatment regime based on the need to improve clinical outcomes in immunodeficient patients and to minimize risks to infection control and public health.

While immune correlates of protection against COVID-19 infection remain incompletely defined, therapeutic strategies involving the transfer of functional antibodies, such as convalescent plasma (CP), to seronegative individuals with severe disease were initially proposed due to its success in the Ebola virus outbreak [8] and in the context of the 2003 severe acute respiratory syndrome (SARS) coronavirus epidemic [9, 10]. Indeed, early COVID-19 CP trials showed favourable outcomes [11], especially in patients with common variable immunodeficiency (CVID) [12, 13], but CP has since been withdrawn due to its inefficacy in large trials treating largely immunocompetent patients and a potential association with driving emergence of immune escape viral variants [14, 15]. Increasing attention has been placed on replacing CP with neutralizing monoclonal antibody (nMab) therapy. In addition to intrinsic limitations of CP such as risk of blood-borne infections and varying epitope specificity, mAbs deliver a higher titre of neutralizing antibodies, which is known to correlate with better clinical outcomes [16].

Ronapreve (REGN-COV2), a combination of two high affinity monoclonal human IgG1 anti-SARS-CoV-2 mAbs casirivimab and imdevimab, is one such mAb cocktail and was authorised in the UK in August 2021 for use in COVID-19 treatment for hospitalised patients with negative (or low level) SARS-CoV-2 Spike (S) antibody. Developed to optimize anti-viral activity against naturally varying viral antigens [17], the antibodies bind to non-overlapping epitopes of the receptor binding domain of the SARS-CoV-2 spike glycoprotein to inhibit viral entry [18]. Promisingly, Ronapreve conferred clinical improvement and rapid viral clearance when administered in combination with remdesivir [19], and was shown to reduce 28-day mortality in seronegative patients hospitalised with COVID-19 in the landmark RECOVERY randomised controlled trial [20].

From December 2021, the NHS authorised additional COVID-19 treatments for the highest risk non-hospitalised patients. This policy enabled use of another nMab, Sotrovimab, or oral antiviral treatments (Molnupiravir or Paxlovid) to be prescribed to non-hospitalised patients with COVID-19 in clearly defined high risk groups [21]. Treatment is delivered by hubs known as Covid Medicine Delivery Units (CMDUs) across the country within 5–7 days of symptom onset and with a positive COVID PCR or lateral flow test.

The nMab Sotrovimab targets an epitope of the receptor binding domain of the spike protein. Data from the COMET-ICE trial demonstrated reduction in hospitalisation and death in patients with mild to moderate COVID-19 treated with Sotrovimab [22]. Sotrovimab is active against BA.1 and BA.1.1 variants and therefore was the first line treatment offered when these variants were dominant. After BA.2 became the dominant circulating subvariant, the antiviral nirmatrelvir/ritonavir (Paxlovid) has been used as first line treatment via CMDUs unless there are contraindications [23]. Anti-viral treatments Molnupiravir (a ribosomal dependent RNA polymerase (RdRp) inhibitor which drives viral mutagenesis) and nirmatrelvir/Ritonavir (Paxlovid; a ritonavir-boosted 3C-protease inhibitor) have been demonstrated to reduce hospitalisation and death in patients with mild to moderate infection in the MOVe-Out and EPIC HR trials respectively [24, 25].

Despite promising immediate clinical outcomes with the use of Ronapreve, sotrovimab, molnupiravir and nirmatrelvir/ritonavir, it is currently unclear whether sustained viral clearance is achieved in immunodeficient patients. Although, persistent SARS-CoV-2 PCR positivity following infection is recognised in immunocompetent hosts and may be of limited significance, the same cannot be assumed for immunodeficient patients at risk of chronic or relapsing infection [2]. We therefore aimed to establish the long-term virological outcomes in the immunodeficient patients treated at our centre.

## Methods

### Patient Cohorts and Data Extraction

An oversight committee was established at the Royal Free London NHS Foundation Trust to monitor inpatient usage of casirivimab-imdevimab (Ronapreve) between September and December 2021. Regular polymerase chain reaction (PCR) tests were performed by healthcare workers during the patients’ admission, as per the UK commissioning guidance. It was also agreed by the oversight committee that immunodeficient patients should be asked to submit regular nasopharyngeal swab and, if possible, sputum samples after discharge from hospital as part of their routine clinical care and these were posted to the individuals’ homes until sustained viral clearance (see below) was achieved. They were also referred to clinical immunology services for further investigation.

Subsequently, immunocompromised patients under the care of the Royal Free London Immunology department with COVID-19 infection between 1 December 2021 and 31^st^ March 2022 who either received no treatment or were treated via CMDUs or other centres, were also sent nasopharyngeal swabs and sputum pots to monitor for sustained viral clearance. Patients were asked to provide sputum samples if they were able to spontaneously expectorate.

Sustained viral clearance was defined as 3 consecutive negative samples regardless of when these were obtained. If the first of these negative samples was obtained within 28 days, patients were categorised as having early sustained viral clearance. If any positive samples were obtained beyond 28 days, patients were classified as having persistent SARS-CoV-2 PCR positivity. If these patients subsequently achieved viral clearance, they were classified as having delayed viral clearance. Some patients had insufficient data for accurate classification. For the outpatient cohort, if date of treatment was not known or no treatment was received, we used the date of first positive test as a baseline. 

As per standard clinical practice, samples with sufficient viral load were sent for sequencing at Public Health England and successful results were deposited as part of COVID-19 Genomics UK (COG-UK) and available on GISAID (accessions provided in the Supplementary Table 1). Consensus level mutations were compared between samples in the dataset and with other publicly available sequences at GISAID. Analyses were performed using the AudacityInstant and CoVsurver mutations Apps within GISAID. Immune escape mutations were retrieved on 13 November 2022 from the list available at: https://people.ucalgary.ca/~gordonp/monoclonal_antibody_serial_passage_escape-S.html.

Baseline data for immunoglobulin levels and lymphocyte subset cell counts (where available) were retrospectively collected for all patients prior to their COVID-19 infection. If data were not available prior to infection, laboratory data were obtained from blood tests taken on admission to hospital in the cohort that received Ronapreve.

The data collection protocol was approved by the Royal Free London COVID-19 oversight committee. Data were only derived from information collected as part of routine clinical care, were processed by the team providing direct care and are presented in fully anonymised form. No specific ethical approval is required for this collation or publication in line with National Health Service Health Research Authority guidance.

### Statistics

Continuous variables were compared between groups using unpaired *t* tests for parametric data or Mann-Whitney tests for non-parametric data (immunoglobulin levels). Categorical variables were compared using chi-squared tests. All *p* values are two-sided and *p* = 0.05 was interpreted as significant. Statistical analysis was performed using GraphPad Prism version 8 or 9.

## Results

### Patient Demographics and Clinical Data

Table 1 presents demographic and clinical data for both cohorts of patients (i.e. those treated with Ronapreve in hospital (*n* = 28) and those who mostly received other treatments (*n* = 75)). Most patients had been vaccinated according to the recommended schedule although we note a relative over-representation of unvaccinated patients in the hospitalised Ronapreve cohort (7 of 28). Three patients in the other cohort were unvaccinated. The most common underlying immune deficiencies were primary or secondary causes of hypogammaglobulinemia. Most patients in the ‘other treatment’ cohort, but only a minority of the Ronapreve cohort, were on immunoglobulin replacement therapy prior to COVID-19 diagnosis.

**Table 1:** Demographic and clinical data on included patients

	Casirivimab-imdevimab cohort (*n* = 28)	Other treatment cohort (*n* = 75)
Mean age (range)	59.6 (38–91)	48.13 (19–85)
Number of male patients (%)	12 (42.9%)	36 (48%)
Ethnicity		
White	18 (64.3%)	51 (68.0%)
Asian (including Indian, Chinese)	4 (14.3%)	3 (4.0%)
Black-African	2 (7.1%)	1 (1.3%)
Other	3 (10.7%)	2 (2.7%)
Not stated	1 (3.6%)	18 (24%)
COVID-19 vaccination status		
Unvaccinated	7 (25.0%)	3 (4.0%)
1 dose received	1 (3.6%)	0 (0.0%)
2 doses received	13 (46.4%)	7 (9.3%)
3 doses received	6 (21.4%)	25 (33.3%)
4 doses received	N/A	27 (36.0%)
Status unknown	1 (3.6%)	13 (17.3%)
Diagnosis		
Primary immune deficiency (PID)	5 (17.9%)	55 (73.3%)
CVID	3 (10.7%)	28 (37.3%)
XLA	0	4 (5.3%)
Other^a^	2 (7.1%)	23 (30.7%)
Haematological malignancy^b^	11 (39.3%)	15 (20.0%)
^Number of patients given anti-CD20^	3 (10.7%)	13 (17.3%)
Other secondary hypogammaglobulinemia^c^	8 (28.6%)	4 (5.3%)
^Number of patients given anti-CD20^	6 (21.4%)	1 (1.3%)
Post-transplant immunosuppression	3 ^di^ (10.7%)	1 ^dii^ (1.3%)
Others^e^	1 (3.6%)	0 (0.0%)
On immunoglobulin replacement		
IVIG	4 (14.3%)	41 (54.7%)
SCIG	2 (7.1%)	23 (30.7%)
None	22 (78.6%)	11 (14.7%)

^a^NF-kappaB2 gain of function (*n* = 1), Specific antibody deficiency (*n* = 3), Undefined hypogammaglobulinemia (*n* = 6), Undefined combined immunodeficiency (*n* = 1), STAT3 gain of function (*n* = 2), Good Syndrome (*n* = 1), IgG subclass deficiency (*n* = 2), DiGeorge (*n* = 4), CD40L deficiency (*n* = 1), IPEX (*n* = 1), Compound heterozygous ICOS mutation (*n* = 1)

One CVID patient also had chronic lymphocytic leukaemia

^b^Chronic lymphocytic leukaemia (*n* = 7), Lymphoma (*n* = 16): {Splenic marginal zone (1); Follicular lymphoma (7); non-Hodgkin’s lymphoma (1); Hodgkin’s lymphoma (1); unspecified (1); Lymphoplasmacytic lymphoma (3); Burkitt’s lymphoma (1), Extra nodal marginal zone lymphoma (1)}, Multiple myeloma (*n* = 2), Waldenstrom’s (*n* = 1)

^c^ANCA-positive vasculitis (*n* = 2); Relapsing remitting multiple sclerosis (MS) (*n* = 1); Takayasu arteritis (*n* = 1); Granulomatosis with polyangiitis (*n* = 1); Rheumatoid arthritis (*n* = 1); On ocrelizumab treatment for MS (*n* = 2); SLE (2); MGUS (*n* = 2)

^di^Combined liver and kidney transplant (*n* = 1), kidney transplant (*n* = 2)

^dii^Transplanted PNP deficient SCID

^e^On mycophenolate mofetil treatment for neuromyelitis optica

Additional clinical data on the Ronapreve-treated cohort are provided in Table 2. Despite the majority having received vaccination, and a reasonable duration between symptom onset and Ronapreve administration, anti-SARS-CoV-2 spike antibody levels were undetectable or very low (notably 4 of 5 patients with detectable levels were receiving immunoglobulin replacement therapy prior to admission). Many patients received additional therapies for COVID-19, most commonly remdesivir.

**Table 2:** Additional clinical information on patients included in casirivimab-imdevimab cohort

Anti-SARS-CoV-2 antibody titre [Detectable range 0.8–2500 IU/mL] (*n* = 28)	
Undetectable	23 (82.1%)
Detectable (Range: 1.04–148.0 IU/mL)	5 (17.9%)
Patients treated with other medication in combination with Ronapreve (*n* = 28)	
Remdesivir	21 (75.0%)
Dexamethasone	11 (39.3%)
Tocilizumab	5 (17.9%)
Sarilumab	4 (14.3%)
Antibiotics*	13 (46.4%)
Timing of Ronapreve administration	
Mean number of days from first positive PCR test to Ronapreve administration [range] (*n* = 28)	19 [0–43]
Mean number of days from symptom onset to Ronapreve administration [range] (*n* = 19)	43 [2–83]

*Co-amoxiclav (*n* = 5), Clarithromycin (*n* = 2), Doxycycline (*n* = 2), Levofloxacin (*n* = 1), Amoxicillin (*n* = 1), Tazocin (*n* = 1), Meropenem (*n* = 1)

Table 3 lists the additional treatments received by patients in the ‘other treatment’ cohort. We note that at least two patients were hospitalised elsewhere, both received remdesivir and one received casirivimab/imdevimab.

**Table 3:** Summary of treatments received in other treatment cohort

Treatment	Number of patients (*n* = 75)
Molnupiravir	3 (4.0%)
Nirmatrelvir/ritonavir	8 (10.7%)
Sotrovimab	31 (41.3%)
Remdesivir	1 (1.3%)
Casirivimab/imdevimab + remdesivir	1 (1.3%)
Unclear	9 (12%)
Nil	22 (29.3%)

**Fig. 1:**
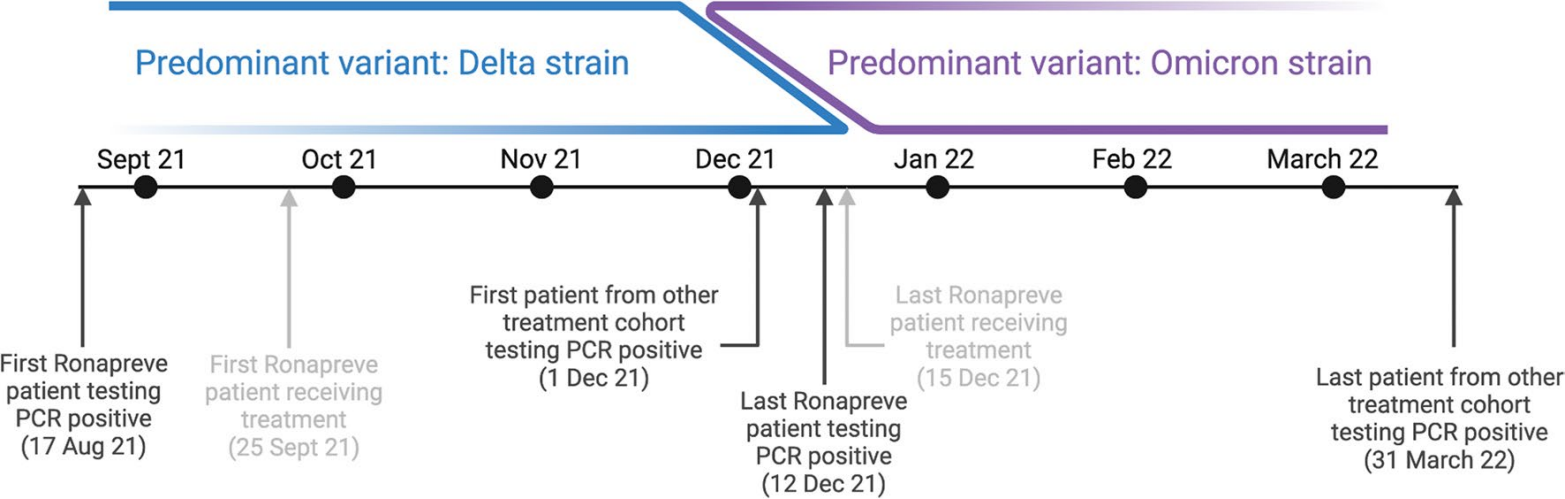
Schema detailing dates of positive tests and treatments in relation to the dominant circulating SARS-CoV-2 variant

Figure 1 shows the timelines of both cohort’s positive tests in relation to the dominant circulating SARS-CoV-2 variants.

### Virological Outcomes

**Fig. 2:**
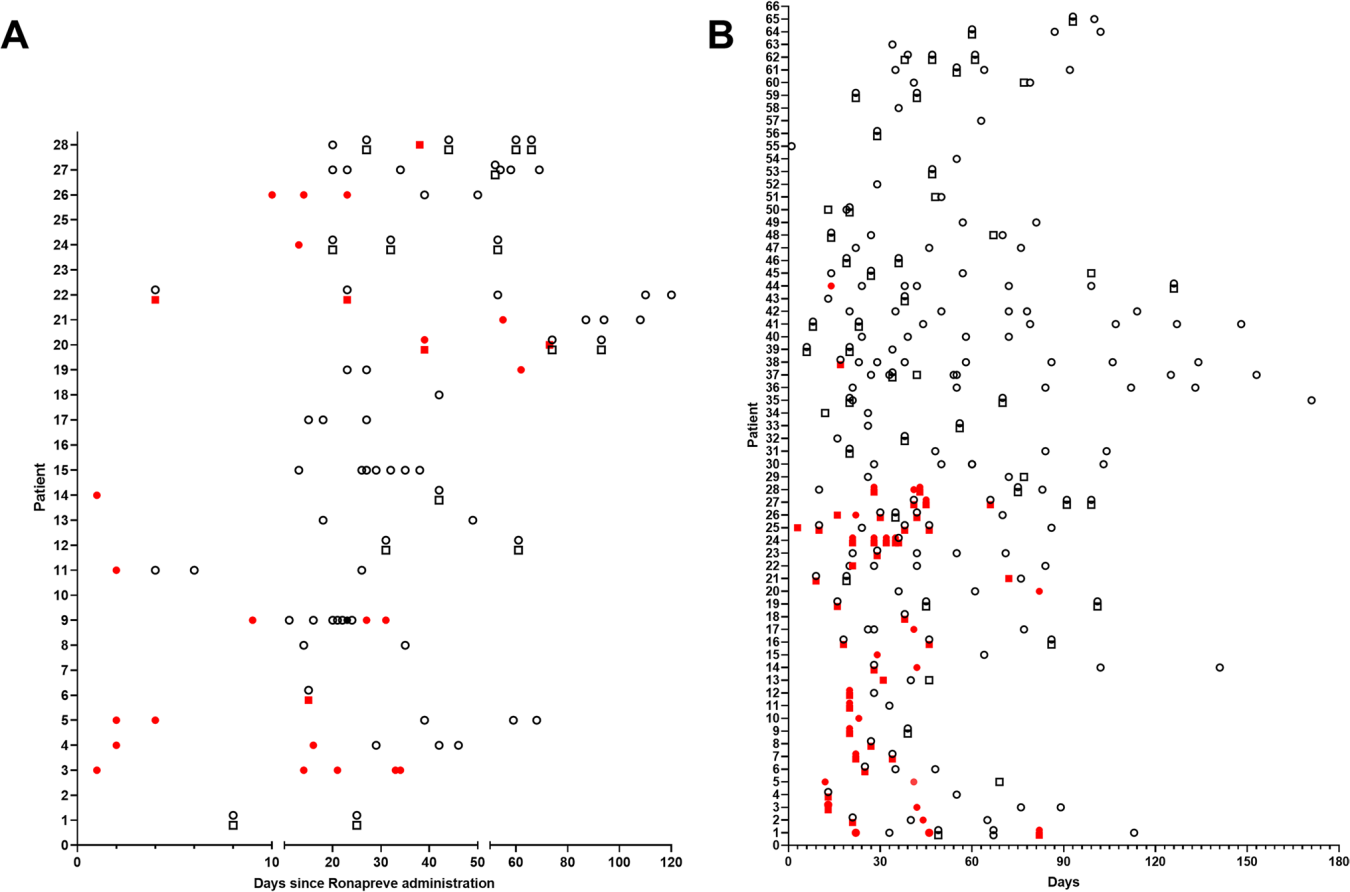
Dot plot summary of virological outcomes in the Ronapreve cohort (**A**) and in the other treatment cohort (**B**). Each patient is presented in one row and patients are numbered in no particular order. Each data point represents one sample. Nasopharyngeal swab samples are represented by circles and sputum samples by squares. Negative samples are white with a black border and positive samples are red. Day 0 is defined as the day of treatment or the day of first positive SARS-CoV-2 PCR result if treatment date is unavailable. Patients were not included if they submitted no follow-up samples

PCR results over time for patients in each cohort are summarised in Fig. 2. We observed sustained viral clearance (at least 3 consecutive negative samples) in 71 of 103 patients (11/28 in the Ronapreve cohort and 60/75 in the other treatment cohort). Among the patients where we did not document viral clearance, 6 died and the others did not submit sufficient samples but we note that in 5 cases, the most recent sample was positive (and this was >28 days from treatment in 3 cases).

We noted sputum positivity despite negative NPS in 25 samples (28.1% of samples where both were submitted contemporaneously). We also observed recurrence of detectable SARS-CoV-2 following at least one negative sample of the same type (i.e. sputum or nasopharyngeal swab) in 12 cases.

We then divided the patients across both cohorts into those who cleared within 28 days and those with persistent PCR positivity beyond this time point.

### Delayed Viral Clearance is Associated with Lower B Cell Counts and Immunoglobulin Concentrations

We interrogated the available data for differences between those patients who were cleared rapidly versus those who did not (Table 4). Age, sex, diagnosis and vaccination status were similar, although we note that 4 of 5 unvaccinated patients had persistent PCR positivity beyond 28 days. We compared treatments received in the outpatient cohort but did not observe any differences, with sotrovimab being used most commonly. Among patients who received sotrovimab, 13/23 (56.5%) cleared within 28 days and 10/23 (43.5%) had persistent PCR positivity beyond 28 days. Among patients who received no treatment, 6/12 (50%) cleared rapidly.

**Table 4:** Comparison of patients who cleared SARS-CoV-2 within 28 days versus those with persistent PCR positivity

	Cleared within 28 days (*n* = 28)	Persistent post-28 days (*n* = 25)	*P* value
Mean (range) age	51.1 (19–85) years	51.4 (19–89) years	0.82
Sex	17F:11M	10F:15M	0.13
Diagnosis			0.36
Primary immune deficiency (PID)	18 (52.9%)	16 (47.1%)	
CVID	9 (50.0%)	9 (50.0%)	
XLA	1 (25.0%)	3 (75.0%)	
Other	8 ^ai^ (66.7%)	4 ^aii^ (33.3%)	
Haematological malignancy	8 (50.0%)	8 (50.0%)	
Number of patients given anti-CD20	6 (54.5%)	5 (45.5%)	
Other secondary hypogammaglobulinemia	2 (100.0%)	0 (0.0%)	
Number of patients given anti-CD20	2 (100.0%)		
Post-transplant immunosuppression	0 (0.0%)	1 (100.0%)	
COVID-19 vaccination			0.10
Unvaccinated	1 (20.0%)	4 (80.0%)	
<4 vaccines	12 (46.2%)	14 (53.8%)	
4+ vaccines	13 (72.2%)	5 (27.8%)	
Treatment (non-Ronapreve cohort only)			0.50
No treatment	6 (50.0%)	6 (50.0%)	
Sotrovimab	13 (56.5%)	10 (43.5%)	
Molnupiravir	0 (0.0%)	2 (100.0%)	
Nirmatrelvir/ritonavir	1 (50.0%)	1 (50.0%)	
(Unknown)	2 (100%)	0 (0.0%)	
Mean (SD) B cell count (×10^9^/L)	0.22 (0.28)	0.06 (0.10)	**0.015**
Mean (SD) CD4+ T cell count (×10^9^/L)	0.59 (0.34)	0.43 (0.22)	0.07
Mean (SD) CD8+ T cell count (×10^9^/L)	0.38 (0.24)	0.43 (0.36)	0.52
Median (IQR) IgG (g/L)	8.10 (6.73–10.65)	8.25 (4.90–10.58)	0.43
Median (IQR) IgA (g/L)	0.40 (0.00–0.95)	0.00 (0.00–0.15)	**0.001**
Median (IQR) IgM (g/L)	0.35 (0.10–1.10)	0.05 (0.00–0.28)	**0.005**

^ai^Specific antibody deficiency (*n* = 2), Undefined hypogammaglobulinaemia (*n* = 3), STAT3 gain of function (*n* = 1), IgG subclass deficiency (*n* = 1) , Digeorge syndrome (*n* = 1)

^aii^NF-kappaB2 gain of function (*n* = 1), Good’s Syndrome (*n* = 2), DiGeorge syndrome (*n* = 1)

**Fig. 3:**
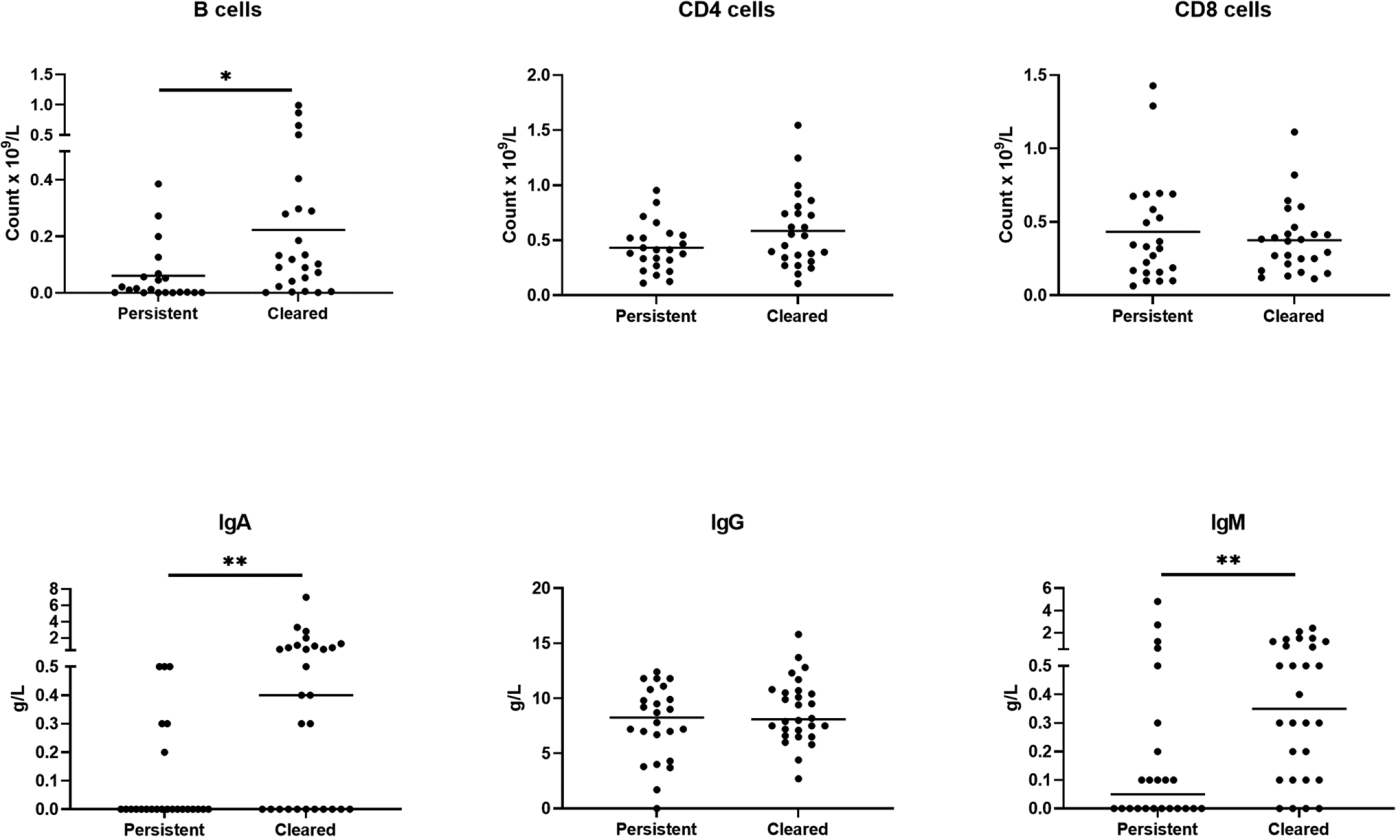
Evaluation of patients who achieved clearance of SARS-CoV-2 within 28 days (*n* = 28) versus those with persistent positive PCR beyond 28 days (*n* = 25). Scatterplot graphs present peripheral B cell (CD19+) counts (**A**); peripheral CD4+ (**B**) and CD8+ T cells (**C**); serum IgA (**D**), IgG (**E**), and IgM (**F**) levels. Each data point represents one patient. Graphs show individual data points and lines represent mean (**A**–**C**) or median (**D**–**F**). Significance was determined using unpaired *t* test (**A**–**C**) or Mann-Whitney *U*-test (D–F). *p < 0.05, **p < 0.01

We observed lower B cell counts in the group with persistent PCR positivity (mean (SD) 0.06 (0.10) ×10^9^/L vs 0.22 (0.28) ×10^9^/L, *p* = 0.015) as well as lower IgA (median (IQR) 0.00 (0.00–0.15) g/L vs 0.40 (0.00–0.95) g/L, *p* = 0.001) and IgM (median (IQR) 0.05 (0.00–0.28) g/L vs 0.35 (0.10–1.10) g/L, *p* = 0.005); Figure 3. There were no differences in CD4+ or CD8+ T cell counts.

### Viral Sequencing does not Reveal Significant Divergence from Circulating Strains

We sought to compare the consensus sequences of the viruses from the patients within these cohorts to determine if certain mutations were unexpectedly over-represented and might be contributing to viral persistence, but found that there were none.

We also compared these sequences with those of other publicly available consensus sequences in GISAID. From this analysis, we observed that 90% of the samples sequenced were identical to at least one other sequence that was circulating previously, with the others having normally just one mutation and a maximum of four novel mutations.

We then sought to analyze if these novel mutations were known to be associated with the immune escape or were in a region of the viral genome which would affect the ability of these patients’ immune system to eliminate with the virus. We did not find any clear association between the variants and immune escape. However, we note that sequencing was not available for many samples, especially those at later time-points where the viral load was generally too low.

### Deaths Within the Cohort

Five deaths occurred in the Ronapreve cohort, with a mean age of 73.4 (4 female, 1 male). Two of these patients had chronic lymphocytic leukaemia, one had myeloma, one had received solid organ transplant and one was on mycophenolate mofetil for neuromyelitis optica. Four of these patients had received 2 or more doses of the vaccine and one was unvaccinated. None were receiving immunoglobulin replacement therapy prior to admission. Three patients died within 2 days of receiving Ronapreve treatment, while two patients died 22 and 34 days after receiving Ronapreve respectively; of note, their follow-up samples remained persistently positive during this period. One death occurred in the non-Ronapreve cohort. This patient had a diagnosis of 22q deletion syndrome and had received 2 doses of the COVID vaccine prior to infection. He was treated with dexamethasone and remdesivir and died within 3 days of receiving treatment.

## Discussion

We have here demonstrated that persistent SARS-CoV-2 PCR positivity beyond 28 days is common in immunodeficient (predominantly antibody deficient) patients despite receipt of currently available treatments. Unlike in immunocompetent hosts where persistent detection of viral RNA is unlikely to represent ongoing viral replication, in immunocompromised people, the virus may still be cultured several months after infection (1). The rapid clearance of PCR positivity corresponding with clinical improvement upon receipt of effective combination therapy in immunocompromised patients with persistent or relapsing disease (2) also suggests that positive results represent viable virus rather than simply shedding of RNA. Consequently, this phenomenon has potential importance from the perspective of individual patient care, infection control and public health.

Clinically favourable outcomes with Ronapreve administration as part of treatment for COVID-19 infection in immunocompromised patients have been reported in B-cell–depleted individuals [26, 27] and we have previously reported that the combination of high-dose (8 g) casirivimab/imdevimab plus remdesivir was highly effective at achieving viral clearance in immunocompromised patients even with chronic or relapsing infection [2]. However, this was with previous viral variants and it remains unclear whether current treatments — especially given as monotherapy — are effective in clearing SARS-CoV-2 in this patient population at the current time. We note that seven patients in our Ronapreve cohort did not receive adjunctive remdesivir and among these were 2 of the 5 deaths and 2 of 4 cases of viral relapse. It is important to note that this cohort was unwell enough to be hospitalised, and received their treatment relatively late in the course of their illness, which might have impacted their response to treatment.

In the other treatment cohort, receipt of COVID-19 therapeutics did not clearly impact upon viral clearance — albeit treatments were not randomly assigned and there may have been bias favouring certain medications depending on the underlying diagnosis, severity of illness or dominant viral variant.

In addition to persistent PCR positivity, we also observed frequent resurgence of positive samples following negative results, emphasising the importance of monitoring for viral clearance in these patients. We also commonly saw positive sputum samples where contemporaneous nasopharyngeal swab samples were negative and we therefore encourage monitoring of sputum samples where patients can provide them [28].

Persistent viral PCR positivity presents infection control challenges, especially in a group with frequent hospital attendances in clinical spaces with other vulnerable patients. It also raises concerns regarding the evolution of novel viral variants, perhaps especially following the receipt of treatment [29]. In this study, we did not observe the appearance of any escape mutations or large numbers of novel variants that would cause concern in the sequences that we analysed. However, as many samples from later time-points were not sequenced, we are unable to provide a conclusive answer to this question. Further sequencing studies are ongoing to answer this question. Viral culture studies would also be informative for immunocompromised patients with persistent PCR positivity in the community in order to determine appropriate public health interventions.

We observed no clear association between virological outcome of these patients with their vaccination status, although we do note a relative over-representation of unvaccinated patients in the hospitalised casrivirimab-imdevimab cohort and that only one unvaccinated patient definitely became PCR-negative within 28 days. We would therefore continue to strongly recommend vaccination for immunodeficient patients. Instead, we observed that the only significant predictors of viral persistence were peripheral B cell count and serum IgA and IgM levels (IgG concentrations will have been artificially increased by immunoglobulin replacement therapy in many patients, which at the time was unlikely to contain significant quantities of anti-SARS-CoV-2 antibody). We also note that only one immunodeficient patient hospitalised with COVID-19 and requiring Ronapreve therapy had a normal B cell count, where this was measured. This is in line with our previous finding that B cell deficiency or absence is the most consistent finding in patient with chronic or relapsing infection [2]. However, we note that some patients with absent B cells (including 1 patient with X-linked agammaglobulinemia) did achieve viral clearance within 28 days and a positive outcome therefore remains possible even in this cohort.

Importantly, many of the hospitalised patients were not previously known to immunodeficiency services with only 6 of 28 already on immunoglobulin replacement therapy. These patients might have benefitted not only from immunoglobulin replacement but potentially also antimicrobial prophylaxis and special care in relation to COVID-19 avoidance and management. We would advocate that all patients with B cell and/or immunoglobulin deficiency should be further evaluated.

This study did not specifically collect data on clinical outcome. However, most patients remained well and did not suffer significant clinical relapse, in line with other recent reports [28]. It may therefore be that COVID-19 treatments improve clinical outcome in these patient cohorts despite viral persistence. Only a placebo-controlled, randomised trial in this group would be able to resolve this question definitively but there would be ethical issues in withholding potentially effective treatment from such vulnerable patients.

Our report has limitations. Data were collected from routine clinical practice and not according to a strict research protocol. There was heterogeneity in the cohort in terms of diagnosis and comorbidity. Sample collection was not complete and was sometimes delayed. In most cases where viral clearance was not established according to our strict criteria (3 consecutive negative samples), patients had at least one negative result, and in the absence of clinical illness may therefore have been less motivated to continue sending samples. As mentioned above, treatments were not randomly allocated. We do not have formal data on clinical outcome. While we managed to produce high-quality sequences for a number of the patients in this study, most patients only had one sequenced sample, and due to the low viral loads at later time points, the available sample is not always that from the latest time point in the series, which can hamper the detection of mutations arising during the infection.

In summary, although we observed sustained clinical improvement in the majority of immunodeficient patients with COVID-19, regardless of treatment, persistent viral PCR positivity or relapse is common. Lower peripheral B cell count and serum immunoglobulin concentrations are a particular risk. This has important implications for the management of these patients and potentially for the ongoing evolution of SARS-CoV-2. We would recommend that all patients with humoral immune deficiency are followed prospectively after SARS-CoV-2 infection, with serial tests until they achieve at least two (ideally three) consecutive negative results, and further treatment should be considered if there is any relapse of symptoms or radiological changes.

## Supplementary information

ESM 1 (CSV 68 kb)

## Data Availability

Routine sequencing data was deposited as part of COVID-19 Genomics UK (COG-UK) and is available on GISAID (accessions provided in Supplementary Table 1). All other data are contained within the manuscript.
